# TREM2 expression promotes liver and peritoneal M2 macrophage polarization in mice infected with *Schistosoma japonicum*

**DOI:** 10.1111/jcmm.17842

**Published:** 2023-07-10

**Authors:** Dandan Zhu, Min Huang, Pei Shen, Bei Zhang, Guo Chen, Jinling Chen, Lian Duan, Yinong Duan

**Affiliations:** ^1^ Department of Pathogen Biology Medical School of Nantong University Nantong People's Republic of China; ^2^ Department of Laboratory Medicine Affiliated Hospital of Nantong University Nantong People's Republic of China; ^3^ Department of Medical Informatics Medical School of Nantong University Nantong People's Republic of China

**Keywords:** macrophage, polarization, *Schistosoma japonicum*, triggering receptor expressed on myeloid cells 2

## Abstract

Schistosomiasis is a tropical parasitic disease that damages the liver and poses a serious threat to human health. Macrophages play a key role in the development of liver granulomas and fibrosis by undergoing polarization from M1 to M2 type during schistosomiasis. Therefore, regulating macrophage polarization is important for controlling pathological changes that occur during this disease. Triggering receptor expressed on myeloid cells 2 (TREM2) expressed on the surface of macrophages, dendritic cells and other immune cells has been shown to play a role in inhibiting inflammatory responses and regulating M2 macrophage polarization, however its role in macrophage polarization in schistosomiasis has not been investigated. In this study, we confirmed that TREM2 expression was upregulated in the livers and peritoneal macrophages of mice infected with *Schistosoma japonicum.* Moreover, the TREM2 expression trend correlated with the expression of M2 macrophage polarization‐related molecules in the liver tissues of *S. japonicum‐*infected mice*.* Using *Trem2* ^−/−^ mice, we also showed that *Trem2* deletion inhibited *Arg1* and *Ym1* expression in liver tissues*. Trem2* deletion also increased the number of F4/80 + CD86+ cells in peritoneal macrophages of infected mice*.* In summary, our study suggests that TREM2 may be involved in M2 macrophage polarization during schistosomiasis.

## 1 INTRODUCTION

Schistosomiasis is a tropical disease caused by infection with the zoonotic parasite schistosome that poses a serious threat to human health. The main pathological features of this disease are granuloma formation and liver fibrosis.^1^, ^2^ During the course of schistosomiasis, macrophages undergo a phenotypic transformation in response to schistosome worm antigens (SWA) and schistosome egg antigens (SEA). Initially, macrophages are polarized into classically activated macrophages (CAMs, M1 macrophages) in response to SWA.^3^, ^4^ M1 macrophages promote the release of pro‐inflammatory factors such as tumour necrosis factor α (TNF‐α), interleukin 12 (IL‐12) and interleukin 1 (IL‐1), leading to an inflammatory response that induces acute schistosomiasis and liver damage.^5^ Subsequently, macrophages tend to polarize into alternatively activated macrophages (ACMs, M2‐type) following stimulation by SEA.^3^, ^4^ M2 macrophages promote the production of anti‐inflammatory factors, including IL‐10, IL‐4 and transforming growth factor β (TGF‐β), which help to inhibit the inflammatory response during the acute infection phase, resulting in the transition from acute to chronic schistosomiasis.^6^ Suppression of the expression levels of these anti‐inflammatory factors may contribute to the high production of reactive oxygen and nitrogen intermediates and the high expression of nitric oxide synthase 2 (NOS2) in the livers from mice infected with *Schistosoma mansoni*, resulting in a large number of deaths in the acute infection phase of mice.^6^, ^7^ Therefore, the shift from M1 to M2 macrophages plays an important role in the formation of granulomas and fibrosis.^8^, ^9^ However, the underlying mechanism of this macrophage phenotypic transformation remains unclear.

Previous studies have shown that multiple cytokines and receptors are involved in the process of macrophage polarization.^4^, ^9^, ^10^ Among them, NOS2, IL‐1β and TNF‐α are highly expressed in M1 macrophages, while arginase 1 (ARG1), mannose receptor C‐type 1 (MRC1, CD206), and chitinase like 3 (CHIL3, YM1) are expressed in M2 macrophages.^11^, ^12^ In schistosomiasis, Ye et al.^11^ reported that both *Arg1* and *Ym1* expression levels were increased in livers and in peritoneal macrophages from mice infected with *Schistosoma japonicum* for 8 weeks. Zhu et al.^13^ also demonstrated that the dynamic expression levels of *Nos2* and *Arg1* mRNA in peritoneal macrophages from mice infected with *S. japonicum* for various time points indicated the shift of macrophages from M1 to M2 type in schistosomiasis.

Triggering receptors expressed on myeloid cells (TREMs), including TREM1, TREM2 and TREM3, have garnered attention from researchers in recent years.^14^ As cell surface receptors belonging to the immunoglobulin superfamily, TREM2 has been reported to promote M2 polarization of macrophages, while TREM1 tends to induce M1 polarization. For example, Zhang et al.^15^ demonstrated that curcumin up‐regulated TREM2 expression in BV2 cells, contributing to a shift from M1 to M2 polarization of BV2 cells. Xu et al.^16^ reported that cGAMP promoted the expression of TREM2 in mouse BV2 microglia cells, accompanied by the polarization of microglia cells from the M1 to the M2 phenotype. Furthermore, Cheng et al.^17^ demonstrated that TREM1 expression was upregulated in CD11b + myeloid cells and spleen CD11b + cells during the early stages of schistosomiasis, reached a peak at 5 weeks post‐infection, and subsequently declined. They also found that SEA of *S. mansoni* inhibited the expression of TREM1 in the human macrophage cell line J774A.1. Our previous research found that SWA of *S. japonicum* promoted the expression of TREM1, and that interference with TREM1 expression downregulated SWA‐induced polarization of M1‐type macrophages in RAW264.7 mouse macrophage cells.^18^ Therefore, TREM1 may regulate the polarization of M1 macrophages during the course of schistosomiasis. However, it has not been reported whether TREM2 is related to M1 or M2 macrophage polarization in schistosomiasis. We speculate that TREM2 participates in M2 macrophage polarization in schistosomiasis. To test this hypothesis, in this study, we used *Trem2* ^*−/−*^ mice infected with *S. japonicum* to observe the role of TREM2 in macrophage polarization in schistosomiasis. We observed that TREM2 expression was upregulated, similar to that of M2 macrophage polarization‐related molecules, in mice infected with *S. japonicum*. We found that *Trem2* deletion may contribute to the downregulation of *Arg1* and *Ym1* expression in the liver and the upregulation of F4/80 + CD86+ cell number among the total peritoneal macrophages in mice infected with *S. japonicum*.

## 2 METHODS

### 2.1 *Schistosoma japonicum* infection mouse models

B6/JGpt‐*Trem2* ^*em1Cd3332in1*^/Gpt knockout mice (*Trem2* ^−/−^ mice) were established by Gempharmatech Co., Ltd (Jiangsu, China) and wild‐type C57BL/6 mice were purchased from Laboratory Animal Centre of Nantong University. All mice were raised in the barrier environment of the Laboratory Animal Centre at Nantong University. Snails infected with *S.japonicum* were provided by National Institute of Parasitic Diseases, Chinese Centre for Disease Control and Prevention. To establish the *S. japonicum* infection models, mice were infected with cercariae *S. japonicum* (15 ± 2) via abdominal skin exposure. The mice were randomly divided into various groups (*n*≧3) and sacrificed at the indicated time. The liver tissues harvested for RNA extraction were preserved in Trizol (Thermo Fisher Scientific) at −80°C. The liver tissues for western blotting were harvested in a tube directly and preserved at −80°C. The liver tissues for immunofluorescence staining and immunohistochemistry were harvested and immersed in formalin solution at 4°C. Peritoneal macrophages were obtained from mice as Xu et al. reported.^12^

### 2.2 Genotype identification

Mouse tails were obtained and digested in Buffer L containing protease plus from the Mouse Direct PCR Kit (Bimake) at 55°C for 15 min. After protease inactivation at 95°C for 5 min, the supernatant was used as the DNA template for PCR according to the instructions of the Mouse Direct PCR Kit. The primer sequences were provided by Gempharmatech Co., Ltd (Jiangsu, China), detailed in Table 1.

**TABLE 1:** Primers for genotype identification.

Primer names	Sequences
*Trem2*‐5S‐in‐tF1^a^	ATGCCTGTCTCCCAAGAACAGA
*Trem2*‐3S‐in‐tR1^b^	GAAGGAATAGGGAGAATCCAGG
*Trem2*‐5S‐in‐tR1^c^	GTGTGCAAGATGGTTAATGACTG

^a,b^ These primers were paired and the PCR products indicated *Trem2* deletion in mice.

^a,c^ These primers were paired and the PCR products indicated wild‐type mice.

### 2.3 RNA extraction and reverse transcription‐quantitative real‐time polymerase chain reaction (RT‐qPCR)

RNA was extracted from tissue homogenate in Trizol and used as a template for cDNA synthesis using the RevertAid First Strand cDNA Synthesis Kit (Thermo Fisher Scientific). The cDNA was then applied as the template for qPCR which was performed using the TB Green Premix Ex Taq II Kit from TAKARA (Japan). Primer sequences are shown in Table 2.

**TABLE 2:** Primers used in RT‐qPCR.

Primer names	Sequences
m*Gapdh*F^18^	TGGAAAGCTGTGGCGTGAT
m*Gapdh*R^18^	TGCTTCACCACCTTCTTGAT
m*Nos2*F^4^	GGAGCGAGTTGTGGATTGTC
m*Nos2*R^4^	GTGAGGGCTTGGCTGAGTGAG
m*Arg1*F^4^	CAGAAGAATGGAAGAGTCAG
m*Arg1*R^4^	CAGATATGCAGGGAGTCACC
m*Trem2*F	TGCTGGAACCGTCACCATCAC
m*Trem2*R	ACTTGGGCACCCTCGAAACTC
m*Ym1*F^11^	AGAAGGGAGTTTCAAACCTGGT
m*Ym1*R^11^	GTCTTGCTCATGTGTGTAAGTGA
m*Il1β*F^11^	AATGACCTGTTCTTTGAAGTTGA
m*Il1β*R^11^	TGATGTGCTGCTGCGAGATTTGAAG

### 2.4 Western blotting

Protein was extracted from tissues using RIPA buffer (Beyotime) and subjected to sodium dodecyl sulfate‐polyacrylamide gel electrophoresis (SDS‐PAGE). The samples were then transferred to polyvinylidene fluoride membranes (Merck) and incubated with 5% nonfat milk. After blocking and washing, the samples were incubated with primary antibody (AF1729, R&D) at 4°C overnight followed by incubation with horseradish peroxidase (HRP)‐conjugated secondary antibody (Proteintech) at room temperature for 2 h. The ECL kit (Merck) was used for visualization on a chemiluminescent instrument.

### 2.5 Immunofluorescence staining and immunohistochemistry

The obtained liver tissues were immersed in formalin solution for at least 24 h, then paraffin embedded in a multifunctional embedding machine. The tissues were then sliced in a rotary microtome.

For immunofluorescence staining, the tissue sections were dewaxed using xylene and placed in citrate buffer for antigen repair. After endogenous peroxidase was blocked, the tissue sections were incubated with donkey serum followed by an antibody against TREM2 (ab86491, Abcam) and F4/80 (30,325 T, CST). Next, the sections were incubated with the associated secondary antibody (112–605‐062 and 711–545‐152, Jackson) and DAPI. After being sealed with 50% glycerin, the samples were observed and photographed via microscopy (Leica).

For immunohistochemistry experiments, the previously dewaxed and antigen repaired tissue sections were treated using an immunohistochemical kit from DAKO (Denmark). Briefly, samples were treated with peroxidase blocking reagent, an antibody against TREM2 (ab86491, Abcam, USA), Envision FLEX/mouse (linker) reagent, Envision FLEX/HRP reagent, DAB, and haematoxylin in sequence. Finally, the samples were observed and photographed under a microscope (Leica). Dark brown staining areas were considered positive.

### 2.6 Flow cytometry

Peritoneal macrophages were obtained from mice^12^ and cultured in a CO_2_ incubator for 4 h to allow for cell adherence. For in vitro experiments, the cells were stimulated with IL‐4 (20 ng/mL, Peprotech) and IL‐13 (10 ng/mL, Peprotech) for 24 h and harvested for flow cytometry using a Flow Cytometer (ACEA). For this, the cells were incubated with PE conjugated antibody against mouse F4/80 (02922–60, Biogems), PE‐Cy7 conjugated antibody against mouse CD11b (03221–77, Biogems), APC conjugated antibody against mouse CD86 or CD206 (17–0862, 17–2061, eBioscience) and FITC conjugated antibody against mouse TREM2 (ab119852, Abcam). The associated isotype antibodies from Biogems, eBioscience or Abcam were used for detection by flow cytometry.

### 2.7 Statistical analysis

All data are presented as mean ± SEM of at least three independent experiments and analysed in SPSS 20.0 using One‐way ANOVA or Independent Samples *T‐*tests. *p* < 0.05 was considered statistically significant between two groups.

## 3 RESULTS

### 3.1 TREM2 expression is upregulated in livers of mice infected with *Schistosoma japonicum*

**FIGURE 1:**
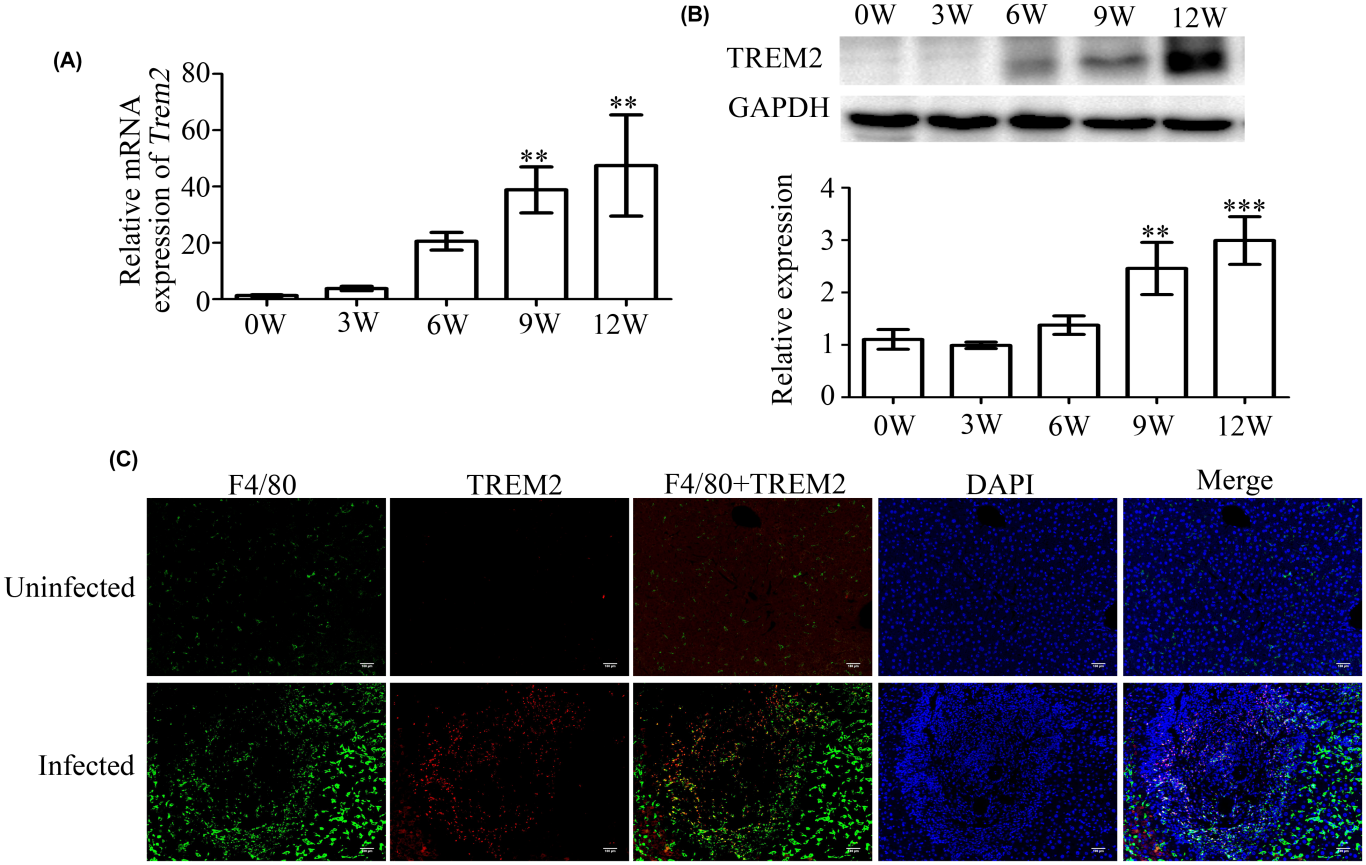
TREM2 expression is upregulated in livers of mice infected with *Schistosoma japonicum*. (A) *Trem2* mRNA expression in livers was detected by RT‐qPCR and compared to uninfected controls. **p < 0.01. (B) TREM2 protein expression in mouse livers was detected by western blotting and compared to uninfected controls. **p < 0.01, ***p < 0.001. (C) The expression of TREM2 in liver tissues of infected (12 W) or uninfected mice was observed by immunofluorescence staining. Green: F4/80+ cells. Red: TREM2+ cells. Yellow: F4/80 + TREM2+ cells. Bar: 100 μm.

We first measured TREM2 expression in the livers of *S. japonicum*‐infected mice and found that *Trem2* mRNA expression was significantly upregulated at 9 and 12 weeks post‐infection compared to uninfected controls (**p < 0.01, Figure 1A). TREM2 upregulation in infected mice was also observed at the protein level via western blotting (Figure 1B). Using immunofluorescence, we also found that the numbers of both F4/80+ and TREM2+ cells were higher in the livers of *S. japonicum‐*infected mice than those of controls (Figure 1C). Notably, F4/80+ TREM2+ cells were also elevated and observed surrounding the egg granuloma in infected livers (Figure 1C). These data suggest that *S. japonicum* infection induces TREM2 expression in mouse livers.

### 3.2 *Arg1* and *Ym1* mRNA expression is enhanced in the liver tissues of mice infected with *Schistosoma japonicum*

**FIGURE 2:**
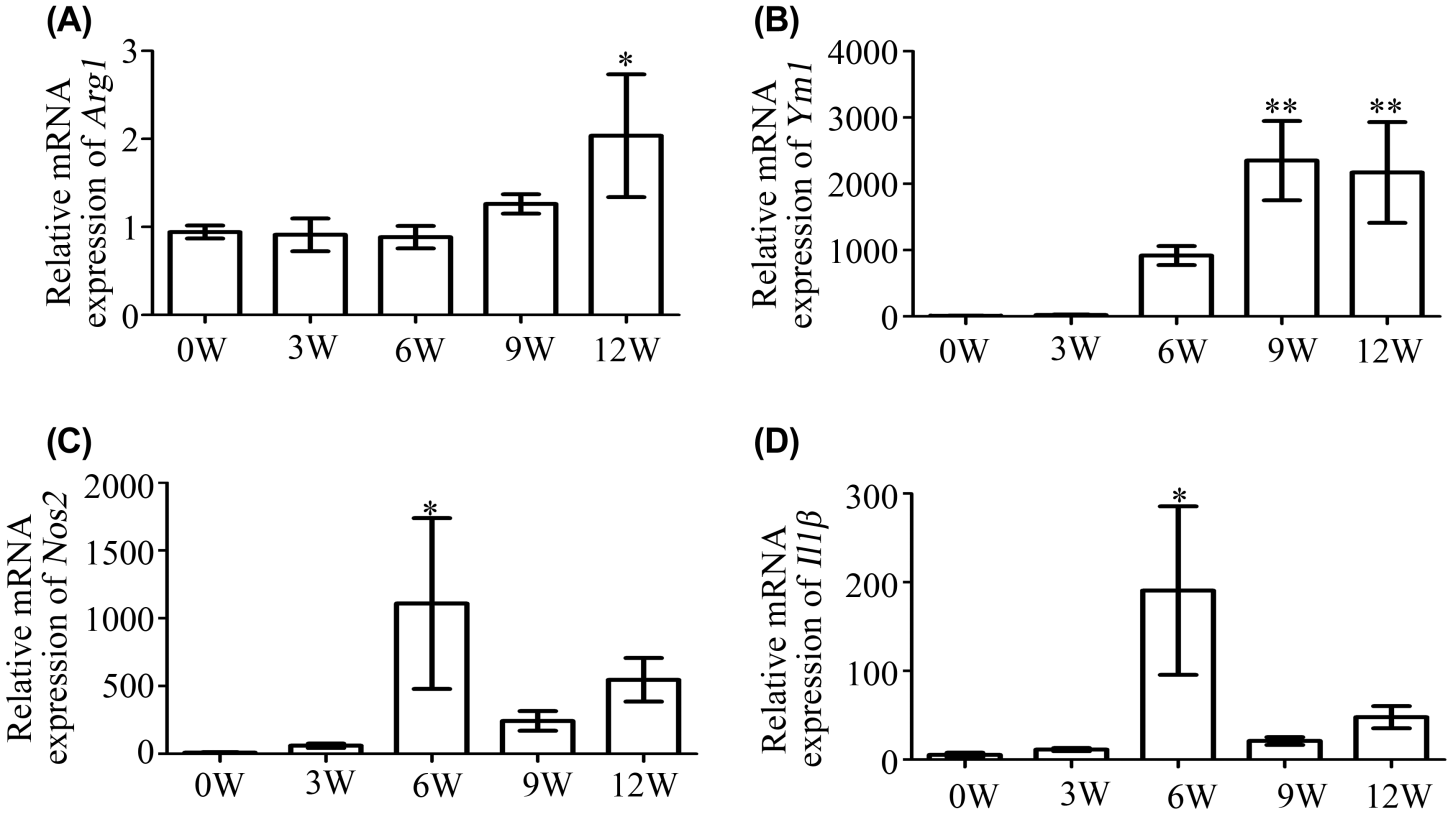
The relative expression of *Arg1* and *Ym1* mRNA is enhanced in the liver tissues of *Schistosoma japonicum‐*infected mice. *Arg1* (A), *Ym1* (B), *Nos2* (C) and *Il1β* (D) mRNA expression levels in livers were detected by RT‐qPCR and compared to expression in uninfected controls. *p < 0.05, **p < 0.01.

Since TREM2 was found to be expressed in F4/80+ cells, we next investigated the expression levels of ARG1 and YM1, two M2 macrophage polarization‐related molecules, in the liver tissues of *S. japonicum‐*infected mice. The results revealed a significant increase in the relative expression levels of *Arg1* mRNA in liver tissues at 12 weeks post‐infection compared with the uninfected controls (*p* < 0.05) (Figure 2A). Additionally, *Ym1* mRNA relative expression levels also significantly increased at 9 and 12 weeks after infection (*p* < 0.01) (Figure 2B). We then examined the expression levels of the M1 macrophage polarization‐related molecules NOS2 and IL‐1β in mouse liver tissues. *Nos2* (Figure 2C) and *Il1β* (Figure 2D) mRNA levels increased upon *S. japonicum* infection, reached a peak at 6 weeks post‐infection (*p* < 0.05), and then decreased. These findings indicate that the expression trend of TREM2 is similar to that of M2 macrophage polarization‐related molecules in the liver tissues of *S. japonicum*‐infected mice.

### 3.3 *Trem2* deletion inhibits *Arg1* and *Ym1* expression in *Schistosoma japonicum‐*infected mouse livers

**FIGURE 3:**
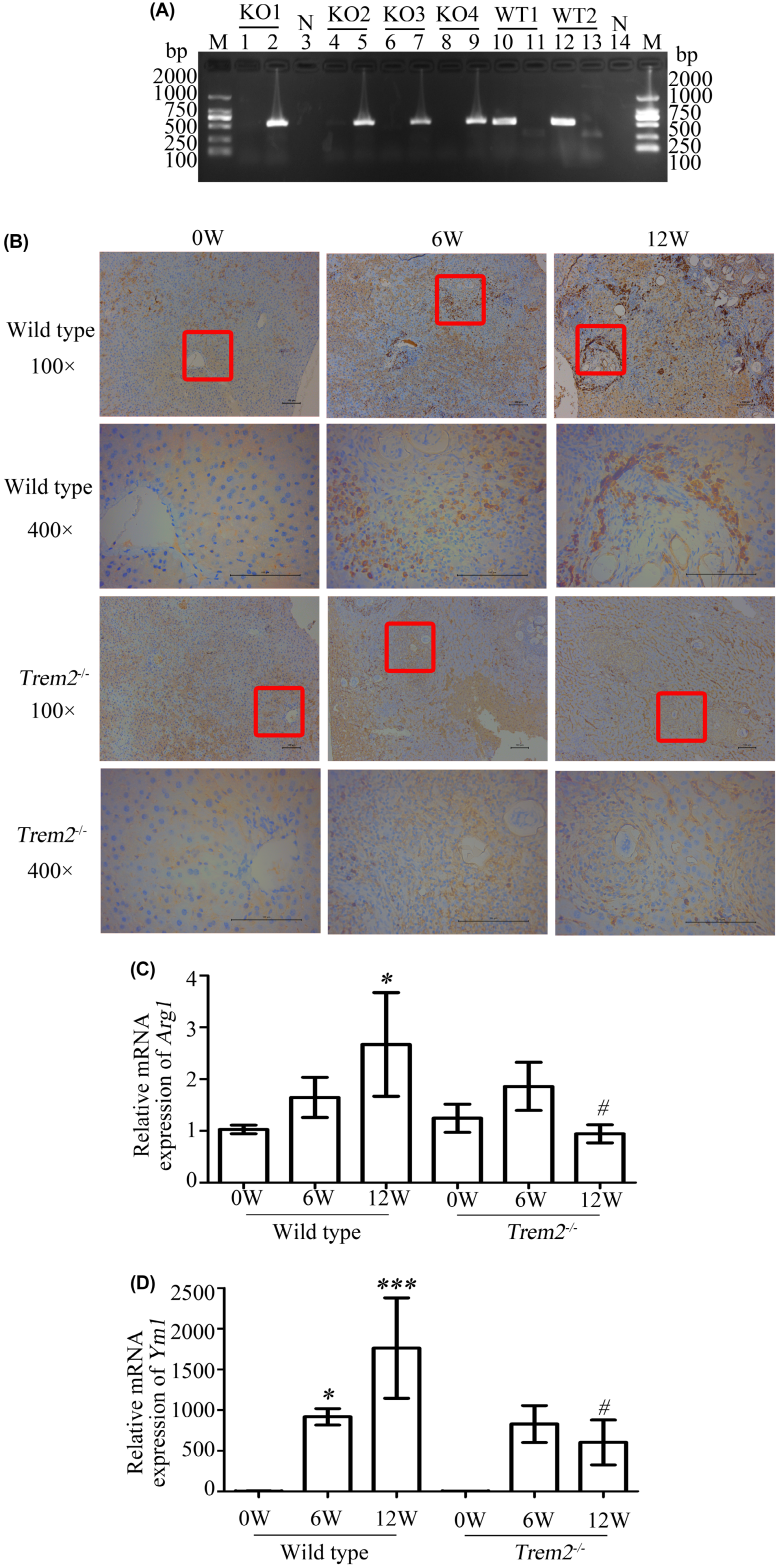
*Trem2* deletion inhibits *Arg1* and *Ym1* expression in livers from mice infected with *Schistosoma japonicum*. (A) Genotype identification was detected by PCR. WT1 and WT2: Two wild‐type mice. KO1 ~ 4: Four *Trem2* ^*−/−*^ mice. N: ddH_2_O was used as negative template DNA. Lanes 1, 3, 4, 6, 8, 10 and 12: Primers (*Trem2*‐5S‐in‐tF1 and *Trem2*‐5S‐in‐tR1) were used and the positive products indicated that this mouse was a wild‐type. Lanes 2, 5, 7, 9, 11, 13 and 14: Primers (*Trem2*‐5S‐in‐tF1 and *Trem2*‐3S‐in‐tR1) were used and the positive products indicated that this mouse was a *Trem2* ^*−/−*^ mouse. (B) TREM2 expression in livers from wild‐type or *Trem2* ^*−/−*^ mice, which were infected with *Schistosoma japonicum* for 6 weeks and 12 weeks, was detected by immunohistochemistry. Bar: 100 μm. (C) and (D) The expression of *Arg1* mRNA and *Ym1* mRNA in livers of wild‐type or *Trem2* ^*−/−*^ mice was detected by RT‐qPCR. Expression was measured relative to the uninfected wild type mice (*p < 0.05, *** *p* < 0.001) or the infected wild type mice after 12 weeks (^#^ *p* < 0.05).

We obtained *Trem2* ^−/−^ (knockout, KO) mice from Gempharmatech Co., Ltd and identified them by PCR. All mice, including KO1, KO2 and KO3, were confirmed as *Trem2* ^−/−^ mice using *Trem2*‐5S‐in‐tF1 and *Trem2*‐3S‐in‐tR1 primers (Figure 3A). In addition, Haematoxylin and eosin staining was also performed to confirm that the wild type mice and *Trem2* ^−/−^ mice were infected with *S. japonicum* successfully (Figure S1).

Immunohistochemistry analysis revealed that TREM2 expression was upregulated in livers from wild‐type mice infected with *S. japonicum* after 6 weeks or 12 weeks, as evidenced by an increase in the positive, dark brown staining area (Figure 3B). However, there was no change in TREM2 expression in the livers of *Trem2* ^−/−^ mice before or after infection (Figure 3B).

At 12 weeks post*‐S. japonicum* infection, *Arg1* mRNA expression was significantly increased in wild type mouse livers compared to that in uninfected controls (*p < 0.05), indicating the presence and increase of M2 macrophage polarization in infected wild‐type mice. This upregulation was absent in *Trem2* ^−/−^ mice infected with *S. japonicum*, as their *Arg1* mRNA levels were significantly lower than those of infected wild type mice after 12 weeks (^#^ *p* < 0.05) (Figure 3C). No significant difference in *Arg1* mRNA levels was observed between the uninfected *Trem2* ^−/−^ mice and the *Trem2* ^−/−^ mice infected with *S. japonicum* at 12 weeks (*p* > 0.05). Furthermore, expression of *Arg1* mRNA, which represents M2 macrophage polarization, did not increase with longer infection duration in *Trem2* ^−/−^ mice. Similar results could be also observed at *Ym1* expression (Figure 3D).

### 3.4 *Trem2* deletion increases the number of F4/80 + CD86+ cells in peritoneal macrophages

**FIGURE 4:**
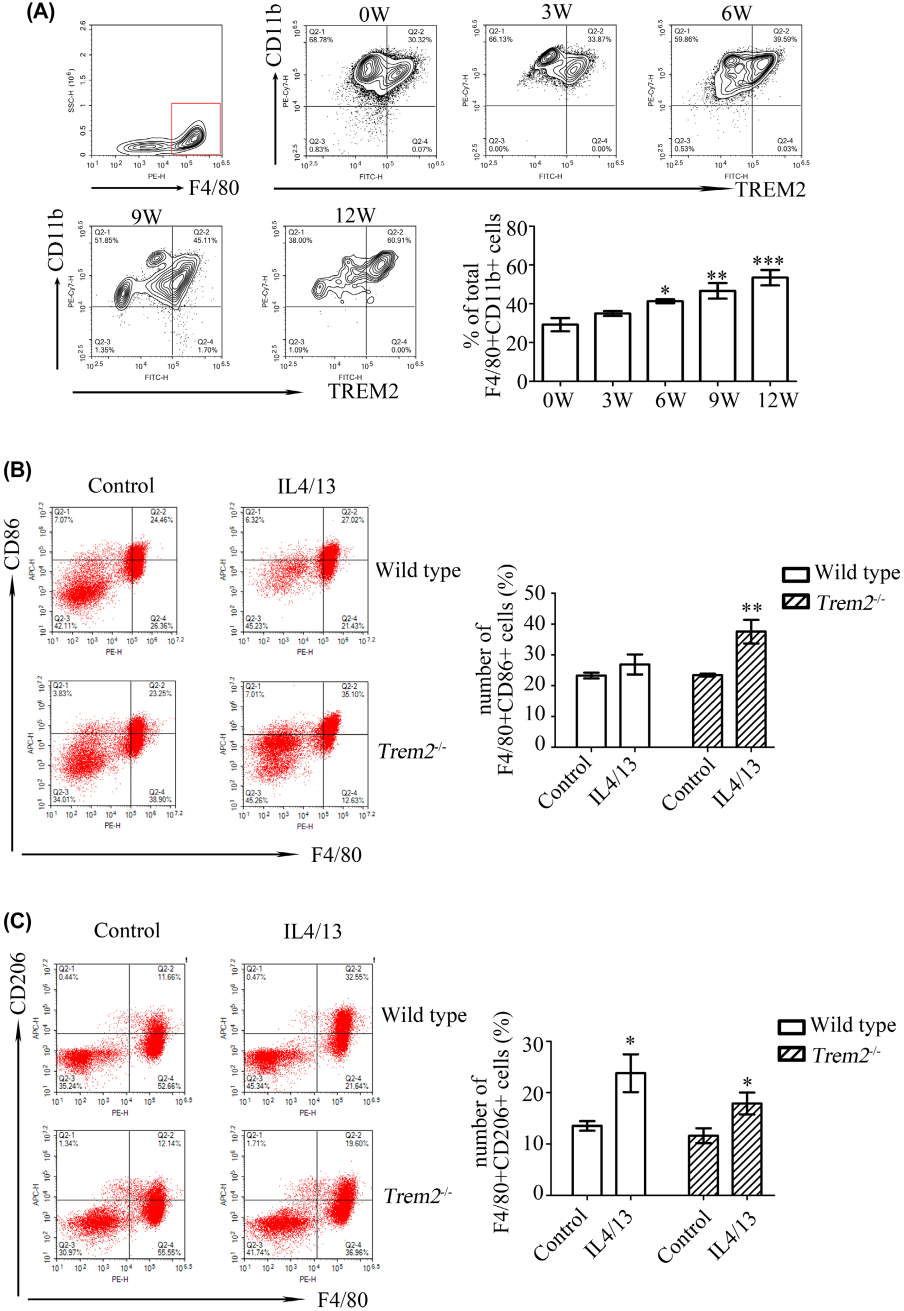
*Trem2* deletion increases F4/80 + CD86+ cells in peritoneal macrophages. (A) TREM2 expression is upregulated in peritoneal macrophages from mice infected with *Schistosoma japonicum*. The number of TREM2+ cells in F4/80 + CD11b + peritoneal macrophages was determined by flow cytometry and compared to uninfected controls. *p < 0.05, **p < 0.01, ***p < 0.001. (B) The number of F4/80 + CD86+ cells in total peritoneal macrophages obtained from wild‐type or *Trem2* ^*−/−*^ mice with or without IL4/13 stimulation was determined by flow cytometry. **p < 0.01. (C) The number of F4/80 + CD206+ cells in total peritoneal macrophages obtained from wild‐type or *Trem2* ^*−/−*^ mice with or without IL4/13 stimulation was determined by flow cytometry. *p < 0.05. All results are relative to their respective controls.

We measured the expression of TREM2 in peritoneal macrophages from mice infected with *S. japonicum* and found that the percentage of TREM2 positive F4/80 + CD11b + cells was enhanced at 6 weeks (*p* < 0.05), 9 weeks (*p* < 0.01) and 12 weeks (*p* < 0.001) post‐infection relative to uninfected controls (Figure 4A). This indicates that TREM2 expression is upregulated in peritoneal macrophages during *S*. *japonicum* infection in mice.

Primary macrophages from wild‐type mice can be polarized to M2 by IL‐4 and/or IL‐13,^19^, ^20^, ^21^ therefore we investigated whether IL‐4 and/or IL‐13 could induce M2 macrophage polarization in *Trem2* ^*−/−*^ mice. We obtained peritoneal macrophages from both wild‐type and *Trem2* ^*−/−*^ mice. We found that the number of F4/80 + CD86+ cells was unchanged when the peritoneal macrophages from wild‐type mice were stimulated with IL‐4 and IL‐13 (IL4/13) (Figure 4B). However, IL4/13 treatment increased the number of F4/80 + CD86+ cells in total peritoneal macrophages obtained from *Trem2* ^*−/−*^ mice (Figure 4B). Moreover, the number of F4/80 + CD206+ cells, which represent M2 macrophages, increased in wild‐type peritoneal macrophages following stimulation with IL4/13 compared to unstimulated controls (Figure 4C, *p* < 0.05). Although IL4/13 also induced an upregulation of F4/80 + CD206+ cells from *Trem2* ^*−/−*^ mice (Figure 4C), this increase was suppressed when the *Trem2* gene was deleted in mouse peritoneal macrophages.

## 4 DISCUSSION

The TREM class of cell surface receptors, including TREM1, TREM2, TREM3, TREML1 and TREML2, belong to the immunoglobulin superfamily and are important for immune regulation and the development of inflammatory diseases.^22^, ^23^, ^24^, ^25^, ^26^ For example, TREM1 triggers and amplifies inflammatory responses and promotes M1‐type macrophage polarization by inducing the expression of pro‐inflammatory factors, while TREM2 promotes the expression of anti‐inflammatory factors through DAP12, thereby inhibiting inflammation and promoting M2‐type macrophage polarization.^24^, ^27^, ^28^, ^29^ Schistosomiasis is known to cause M1‐type macrophage polarization of mouse hepatic macrophages during the acute disease phase, while M2‐type macrophage polarization is observed during the chronic stage.^13^ In our previous study, we confirmed that TREM1 participates in M1 macrophage polarization induced by SWA, however, the role of TREM2 in M2 macrophage polarization during chronic schistosomiasis remained unclear.

Previous studies have highlighted the potential role of TREM2 in the development and progression of liver diseases. Specifically, upregulation of TREM2 expression has been observed in both carbon tetrachloride‐induced acute and chronic liver injury models, in liver tissues of mice following biliary ligation,^30^ as well as in liver tissues of patients with cirrhosis and hepatocellular carcinoma (HCC).^30^, ^31^ In this study, we found that TREM2 expression was also increased in the livers of *S. japonicum*‐infected mice. Interestingly, TREM2+ cells mainly surrounded the egg granuloma and partially co‐localized with F4/80+ cells in the liver. However, we also observed that some TREM2+ cells were not co‐localized with F4/80+ cells, which may be due the fact that TREM2 is expressed not only in F4/80+ cells like macrophages, but also in other F4/80‐ cells such as dendritic cells, hepatic stellate cells and adipocytes.^30^, ^32^ Additionally, the variety of F4/80+ cells may contribute to the discrepancy, as F4/80 + CD86+ cells represent M1 polarized macrophages which express low levels of TREM2, while F4/80 + CD206+ cells represent M2 polarized macrophages which may express higher levels of TREM2. However, further studies are needed to confirm whether macrophages with low TREM2 expression are indeed M1 macrophages. Overall, the increased number of F4/80+ TREM2+ positive co‐staining cells in *S. japonicum*‐infected mouse livers suggests that TREM2 may be involved in the polarization of macrophages during the course of infection, potentially contributing to the M2 polarization of macrophages.^27^, ^33^

Xu et al.^4^ demonstrated that during *S. japonicum* infection, the dominant type of activated peritoneal macrophages shifts from F4/80 + CD16/32+ (M1) macrophages to F4/80 + CD206+ (M2) macrophages. Consistent with this macrophage transformation trend, we found that TREM2 expression in F4/80 + CD11b + peritoneal macrophages gradually increased during *S. japonicum* infection. Importantly, the TREM2 expression trend in mouse livers also correlated with the expression of the M2 macrophage polarization molecules ARG‐1 and YM1. Using *Trem2* ^*−/−*^ mice, we further determined that *Trem2* deletion inhibited *Arg1* and *Ym1* expression in livers of mice infected with *S. japonicum* and increased F4/80 + CD86+ cells among peritoneal macrophages. These results were similar as those reported by Jiawei Zhang et al.^15^ and Xiaobao Zhang et al.^34^ They found that overexpression of TREM2 could promote *Arg1* mRNA expression in microglia and suppress LPS‐induced elevation of TNF‐α and IL‐1β in microglia.^34^ TREM2 may regulate the balance of M1/M2 polarization in microglia induced by curcumin or LPS.^15^, ^34^ Collectively, these results suggest that TREM2 may be involved in M2 macrophage polarization in schistosomiasis.

In conclusion, *S. japonicum*‐infection may induce TREM2 expression in mouse livers and TREM2 may be involved in M2 macrophage polarization during schistosomiasis. However, additional research is required in order to determine the mechanism.

## AUTHOR CONTRIBUTIONS

**Dandan Zhu:** Conceptualization (supporting); data curation (equal); formal analysis (lead); funding acquisition (equal); writing – original draft (lead); writing – review and editing (equal). **Min Huang:** Data curation (equal). **Pei Shen:** Formal analysis (supporting); funding acquisition (supporting); writing – original draft (supporting); writing – review and editing (supporting). **Bei Zhang:** Data curation (supporting). **Guo Chen:** Data curation (supporting). **Jinling Chen:** Resources (supporting). **Lian Duan:** Resources (equal). **Yinong Duan:** Conceptualization (lead); funding acquisition (equal); resources (lead); supervision (lead); validation (lead).

## CONFLICT OF INTEREST STATEMENT

The authors have stated explicitly that there are no conflicts of interest in connection with this article.

## Supporting information


**Figure S1.** The wild type mice and *Trem2* ^−/−^ mice were infected with *Schistosoma japonicum* successfully. Haematoxylin and eosin staining was performed to observe the status on the development of liver granulomas. Bar: 100 μm.

## Data Availability

The data supporting the conclusions of this article are included within the article
