# Supplementary material for: TREM2 expression promotes liver and peritoneal M2 macrophage polarization in mice infected with Schistosoma japonicum
Source: J Cell Mol Med. 2023 Jul 10;27(15):2261–9. doi: 10.1111/jcmm.17842 (PMC10399532; doi:10.1111/jcmm.17842)
Supplement: Supplementary file 1 — Figure S1. The wild type mice and Trem2 −/− mice were infected with Schistosoma japonicum successfully. Haematoxylin and eosin staining was performed to observe the status on the development of liver granulomas. Bar: 100 μm. [file JCMM-27-2261-s001.docx]

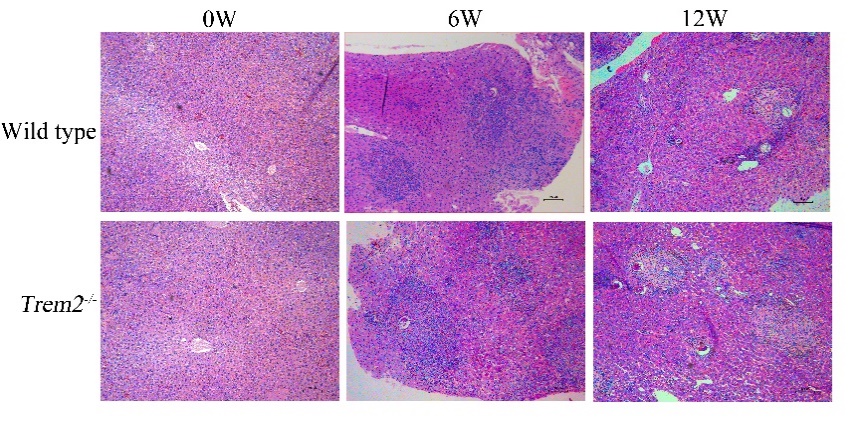


Figure S1. The wild type mice and *Trem2*^-/-^ mice were infected with *Schistosoma japonicum* successfully. H&E staining was performed to observe the status on the development of liver granulomas. Bar: 100 μm.
